# Describing the association between socioeconomic inequalities and cancer survival: methodological guidelines and illustration with population-based data

**DOI:** 10.2147/CLEP.S150848

**Published:** 2018-05-17

**Authors:** Aurélien Belot, Laurent Remontet, Bernard Rachet, Olivier Dejardin, Hadrien Charvat, Simona Bara, Anne-Valérie Guizard, Laurent Roche, Guy Launoy, Nadine Bossard

**Affiliations:** 1 Cancer Survival Group, Department of Non-Communicable Disease Epidemiology, Faculty of Epidemiology and Population Health, London School of Hygiene and Tropical Medicine, London, United Kingdom; 2 Non-Communicable Diseases and Trauma Direction, The French Public Health Agency, Saint-Maurice, France; 3 Department of Biostatistics and Bioinformatics, Hospices Civils de Lyon, Lyon, France; 4 UMR 5558, Biometry and Evolutionary Biology Laboratory, Biostatistics Health Group, CNRS, University Lyon 1, Lyon, France; 5 National Institute of Health and Medical Research U1086 ANTICIPE, Caen, France; 6 Calvados Digestive Cancer Registry, Centre Hospitalier Universitaire, Caen, France; 7 Prevention Division, Center for Public Health Sciences, National Cancer Center, Tokyo, Japan; 8 Manche General Cancer Registry, Centre Hospitalier Public du Cotentin, Cherbourg-en-Cotentin, France; 9 Calvados General Cancer Registry, Centre François Baclesse, Caen, France

**Keywords:** cancer net survival, socioeconomic inequalities, European Deprivation Index, excess mortality hazard, flexible parametric model

## Abstract

**Background:**

Describing the relationship between socioeconomic inequalities and cancer survival is important but methodologically challenging. We propose guidelines for addressing these challenges and illustrate their implementation on French population-based data.

**Methods:**

We analyzed 17 cancers. Socioeconomic deprivation was measured by an ecological measure, the European Deprivation Index (EDI). The Excess Mortality Hazard (EMH), ie, the mortality hazard among cancer patients after accounting for other causes of death, was modeled using a flexible parametric model, allowing for nonlinear and/or time-dependent association between the EDI and the EMH. The model included a cluster-specific random effect to deal with the hierarchical structure of the data.

**Results:**

We reported the conventional age-standardized net survival (ASNS) and described the changes of the EMH over the time since diagnosis at different levels of deprivation. We illustrated nonlinear and/or time-dependent associations between the EDI and the EMH by plotting the excess hazard ratio according to EDI values at different times after diagnosis. The median excess hazard ratio quantified the general contextual effect. Lip–oral cavity–pharynx cancer in men showed the widest deprivation gap, with 5-year ASNS at 41% and 29% for deprivation quintiles 1 and 5, respectively, and we found a nonlinear association between the EDI and the EMH. The EDI accounted for a substantial part of the general contextual effect on the EMH. The association between the EDI and the EMH was time dependent in stomach and pancreas cancers in men and in cervix cancer.

**Conclusion:**

The methodological guidelines proved efficient in describing the way socioeconomic inequalities influence cancer survival. Their use would allow comparisons between different health care systems.

## Introduction

Assessing the relationship between socioeconomic deprivation and cancer survival is important as socioeconomic differences in cancer survival are still observed even in countries with universal health care coverages.^1^–^6^ Describing this relationship at the population level calls for population-based cancer registry data, but the way of performing the analysis is challenging. Indeed, several methodological conditions should be met: 1) the use of a relevant measure of deprivation, usually ecological (ie, defined at an area level) as individual level of deprivation is not routinely collected in population-based data; 2) the use of a relevant mortality indicator such as the excess disease-specific mortality among cancer patients vs noncancer subjects; 3) the use of a regression model able to deal first with nonlinear functional forms of the association between continuous prognostic factors and the excess mortality (eg, between the deprivation index and the excess mortality throughout the range of the deprivation index values) and then deal with time-dependent associations (eg, nonconstant association between the deprivation index and the excess mortality over the time elapsed since diagnosis); 4) the use of an appropriate method for statistical inference that accounts for the statistical dependency between patients who share similar characteristics because they live in the same area across which the ecological deprivation index is defined (this is especially important when the interest lies in estimating regression parameters associated with the ecological deprivation variable);^7^–^9^ and 5) the use of a measure that summarizes the “importance” of the cluster level on the Excess Mortality Hazard (EMH).

Several recent ecological population-based studies addressing the question of the association between social deprivation and cancer survival were found in the literature.^4^–^6^,^10^–^12^ Disease-specific mortality was generally the outcome of interest, usually estimated using an excess mortality approach, while cause-specific mortality approach was also used (using the cause of death). Statistical methods relied on either nonparametric or parametric regression modeling approaches. When modeling approaches were used, the adopted deprivation index was considered as a categorical variable (quintiles). When regression models included the age at diagnosis as a prognostic factor, it was most of the time considered as a categorical variable. Finally, some studies explored the time-dependent associations between the variables and the hazard but it is not the rule, and none had taken into account the hierarchical structure of the data.

The present work proposes methodological guidelines for addressing the above-mentioned challenges (1–5) and illustrates their use through an investigation of the association between socioeconomic deprivation and cancer survival from solid tumor cancers up to 10 years after diagnosis. It is worth noticing that, although the article uses the term “effect” in a few places, no causal association is implied.

## Materials and methods

### Data

We used population-based cancer registry data that cover two contiguous Départements [French administrative areas] of West France (Calvados and Manche, nearly 1.1 million inhabitants). The quality and exhaustiveness of the included reg istries are certified every 4 years by an audit of the National Institute of Health and Medical Research (INSERM), the “Santé Publique France” agency, and the French National Cancer Institute. The incidence data from those registries are regularly included in the “Cancer Incidence in Five Continents” monograph series of the International Agency of Research on Cancer, where their quality and exhaustiveness are also assessed.

We analyzed cancer cases diagnosed between 1997 and 2010 in people aged >15 years at diagnosis. The follow-up of all cases ended on June 30, 2013. The 17 cancers under study are displayed in Tables S1 and S2.

The data from these registries are not publicly available. We analyzed these data under the ethical approval obtained by each registry from the French institute “Commission Nationale de l’Informatique et des Libertés” (“998018” for the Calvados digestive cancer registry, “981001 V1” for the Calvados general cancer registry, and “912669” for the Manche cancer registry).

### The measure of social deprivation

Because individual levels of deprivation are not routinely collected, ecological measures defined at area levels have been proposed.^13^,^14^ These measures are considered as good proxies of individual deprivation in relatively small areas^15^ and measure additionally the patients’ social and economic environment (“contextual variables”).^7^,^16^,^17^

The European Deprivation Index (EDI) was developed using information from the European Union Statistics on Income and Living Conditions (EU-SILC) survey as well as other country-specific information.^18^ The ultimate goal of this index is to have in each European country an ecological deprivation index based on (country-specific) census variables using the same methodological approach for its construction while accounting for cultural and social specificities of each European country. The approach relies on the concept of relative deprivation, first proposed by the sociologist Peter Townsend.^19^ Deprivation refers to unmet fundamental needs caused by the lack of resources of all kinds (not only financial), those fundamental needs differing between societies (thus “relative” as it refers to deprivation specifically for a given society). Individuals can be said deprived when they lack the resources to obtain those types of needs (diet, type of living conditions, amenities, or services), which are obtained by the majority of people in the societies to which they belong to.

The EU-SILC is organized every year in every country of the EU-28. Based on a representative panel of European household, individuals answer some detailed questions on their living condition in each country. The construction of the EDI can be summarized as follows: First, fundamental needs are identified for each European country using the EU-SILC data. Among them, those associated with both objective poverty and subjective poverty are used to build a deprivation indicator at the individual level. Then, after identifying which variables are available at both the individual level (EU-SILC) and the area level (census), the area-level variables that are best correlated with the deprivation indicator built in the previous step are used to finally construct the area-based deprivation index. Details of concepts and construction methods are available in the previous methodologic papers.^18^,^20^

In France, this EDI is assigned to each IRIS (*Îlot Regroupé pour l’Information Statistique*, a geographical area of nearly 2000 individuals); it was then assigned to each patient from a given IRIS. The correspondence between a patient and an IRIS was determined according to the patient’s address at the time of diagnosis. This used a Geographic Information System software (ArcGIS 10.2) and a street map database (BD TOPO premium). In this work, we used the EU-SILC from 2006 to derive the EDI, which ranges for France from −17.3 to 51.1 (quintile 1 [Q1]: [−17.3;−2.9], Q2: [−2.9;−1.4], Q3: [−1.4;0], Q4: [0;2.1], and Q5: [2.1;51.1]).

### The Excess Mortality Hazard

A relevant disease-specific mortality indicator is needed. Cancer-specific mortality using the cause of death is very popular but hardly usable in our context. Actually, the cause of death may be inaccurate or unreliable, especially for long-term studies, because it may be diversely coded over time and between regions. Besides, attributing a single cause of death to elderly people is debatable.^21^ Alternative approaches called “EMH methods” have been then developed;^22^–^26^ these do not require the knowledge of the cause of death.^27^–^30^ The basic idea of EMH methods is comparing the mortality between cancer patients and noncancer subjects with the same sex, age, and other main characteristics. The mortality of cancer-free subjects, called “expected mortality”, is assumed to be correctly given by the general-population mortality, which is a known value. The EMH is then estimated by subtracting the expected mortality from the mortality of cancer patients; it provides the excess mortality due (directly or indirectly) to cancer at any time after diagnosis. For the expected mortality hazards, we used the French population mortality rates by sex, age (0–99 years), Département [French administrative area], and calendar year (1997–2013) as provided by the French Institut National de la Statistique et des Études Économiques.

From the EMH, we derived directly the net survival, using the classical relationship between hazard and survival. Net survival is then the probability of survival of cancer patients if the cancer under study was the only cause of death. In population-based studies, this key indicator allows comparisons between countries or periods and is not affected by differences in mortalities from other causes.^31^

### Regression modeling of the EMH

In cancer patients, the relationship between a prognostic factor (such as EDI) and EMH may be complex.^27^,^32^,^33^ A multivariable regression model has to consider these complex relationships using flexible functions.^22^,^34^ We defined a “full model” that modeled the EMH (on a log scale) as a function of time, age at diagnosis, year of diagnosis, and EDI, with these last three variables having time-dependent coefficients and nonlinear functional forms (thus leading to time-dependent and nonlinear log excess hazard ratios [EHRs], as denoted hereafter).

In addition, the EDI being an ecological variable and the individuals living in a given area sharing similar characteristics (including the EDI variable), a specific statistical method should allow dealing with the hierarchical structure of the data (ie, multilevel data with dependence between individuals at each level).^7^–^9^ This was done by including a normally distributed random effect at the IRIS level.^34^

Thus, in formula, the “full model” for the EMH *λ*_+_ is written as follows:

$$\lambda_{+}(t,a,y,i|w)=\lambda_{0}(t)\exp(g(a)+h(t)a+j(y)+k(t)y+m(i)+n(t)i+w)$$

where *λ*_0_(*t*) is the baseline hazard, *a* the age at diagnosis, *y* the year of diagnosis, *i* the EDI, and *w* the random effect defined at the IRIS level (with mean 0 and standard deviation *σ*). The logarithm of the baseline hazard and the functions *h*, *k*, *n* were modeled with quadratic B-splines with knots located at 1 and 5 years, and the nonlinear functional forms *g*, *j*, *m* were modeled using quadratic splines with one knot (located at 70 years for age at diagnosis, at 2000 for the year of diagnosis, and at 0 for the EDI).

Finally, because the estimated standard deviation of the random effect per se is difficult to interpret, we summarized the “importance” of the cluster level on the EMH using the median excess hazard ratio (MEHR).^35^ This value reflects the influence of the cluster context as a whole, thus measuring the “general contextual effect”.^17^,^35^ The MEHR corresponds to the median relative change in the EMH when comparing identical subjects from two randomly selected different clusters that are ordered by risk.^35^

The analysis was separately conducted in men and women and used the iterative model-building strategy recommended by Wynant and Abrahamowicz.^36^ Starting with the “full model”, this strategy eliminates spurious time-dependent and nonlinear EHR functions of the three variables using the likelihood ratio test and 0.05 as significance threshold. This led to retain a final model for each sex-cancer couple. However, unlike the original proposal,^36^ we kept by default the simplest EHR (ie, linear and time-constant) for each of the three variables.

To implement the advocated statistical methods, we developed a specific package named mexhaz (version 1.1), which runs on R software (version 3.2.0). Both the software and the package may be freely downloaded from the CRAN repository (https://cran.r-project.org/).

### Indicators produced

For each sex-cancer couple, we predicted from the final model the age-standardized net survival (ASNS) at 1, 5, and 10 years after diagnosis per deprivation quintile of the French population using the International Cancer Survival Standard weights.^37^ We used the delta method to derive the 95% confidence intervals (CIs) for the ASNSs assuming the normality of the log of the cumulative excess hazard.

The change in the EMH over the time elapsed since diagnosis was illustrated for three values of age and three values of the EDI: the 10th, 50th, and 90th percentiles of each variable distribution observed in each sex-cancer couple.

When the EDI was retained in the final model with a time-constant coefficient and a linear functional form, we reported the EHR for 1-unit increase of the EDI with its 95% CI. When the EDI was retained in the final model with time-constant coefficients and with a nonlinear form, we plotted the EHR vs the EDI values. When the EDI was retained in the final model with the time-dependent coefficient, we plotted the EHR vs the EDI values at various times after diagnosis. Because the sample size was usually small in this work, we focused on the effect size and its pattern rather than on the statistical significance in interpreting differences in function of the EDI.

For each sex-cancer couple, we calculated the MEHR with and without adjustment on the EDI from the final model to compare the general contextual effect on the EMH.

## Results

### Data description

Tables S1 and S2 display the number of cases and deaths over 10 years after diagnosis. The highest numbers of deaths were found in deprivation quintiles Q4 and Q5 that group the most deprived people. These deaths represent almost 50% of all events in most cancers (Tables S1 and S2). A few sex-cancer couples were not analyzed because of the low number of deaths (<300 in each of esophagus, liver, and larynx cancers in women; breast cancer in men; and thyroid cancer in men and women).

### Deprivation (EDI)

A constant-in-time EHR of the EDI with a linear functional form was retained in most cancer sites, except lip–oral cavity–pharynx (LOCP; nonlinear EHR in both sexes), stomach (time-dependent EHR), pancreas (nonlinear and time-dependent EHR) in men, and cervix uteri (nonlinear and time-dependent EHR; Table S3).

#### Five-year ASNS according to EDI

In men, a substantial difference in 5-year ASNS was seen between deprivation quintiles Q1 (the least deprived) and Q5 (the most deprived) regarding LOCP cancers (41%; 95% CI: [38;43] vs 29% [27;31]). A similar difference was seen regarding skin melanoma (87% [84;89] vs 76% [72;80]; Table 1). The difference in 5-year ASNS between Q1 and Q5 was nearly 7% for colon–rectum and bladder cancers, 6% for kidney, 5% for prostate cancer, 4% for lung and liver cancers, 3% for stomach and larynx cancers, and ≤2% for esophagus and pancreas cancers. Tables S4 and S5 show the results of 1- and 10-year ASNS by deprivation quintile. For pancreas cancer, the absence of the impact of EDI on the 5-year ASNS contrasts greatly with the substantial difference observed in 1-year ASNS (36% [33;40] in Q1 vs 25% [22;28] in Q5) (Table S4). This is due to a special time-dependent EHR of the EDI that we explain later.

In women, as in men, a substantial difference in 5-year ASNS was observed between Q1 and Q5 regarding LOCP cancers, 55% [49;62] vs 43% [39;47], with a higher predicted ASNS for Q2 (60% [56;64]) than Q1. The difference in 5-year ASNS between Q1 and Q5 was around 6% for bladder cancer; 4% for breast and melanoma cancers; 3% for colon–rectum, lung, and ovary cancers; and ≤2% for CNS, pancreas, and stomach cancers. In contrast, for cervix uteri, the 5-year ASNS was lower in the less deprived women in comparison with the most deprived: 55% [51;59] for Q1 vs 64%–66% for the other quintiles. This pattern was also found for 1- and 10-year ASNS (Tables S4 and S5) for that cancer site.

#### Changes over time since diagnosis of the EMH according to EDI and age, and complementary illustrations of the relationship between EDI and EMH

**Figure 1:**
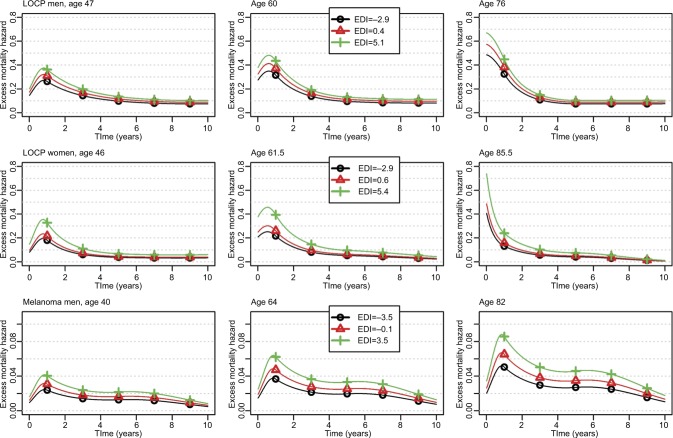
Changes over time since diagnosis of the excess mortality hazard for the 10th, 50th, and 90th percentiles of the age distribution (left, middle, and right column, respectively) and for the 10th, 50th, and 90th percentiles of the EDI distribution (curves with black circles, red triangles, and green crosses, respectively) regarding LOCP in men and women, and melanoma in men; patients diagnosed in 2010. **Abbreviations:** EDI, European Deprivation Index; LOCP, lip–oral cavity–pharynx.

**Figure 2:**
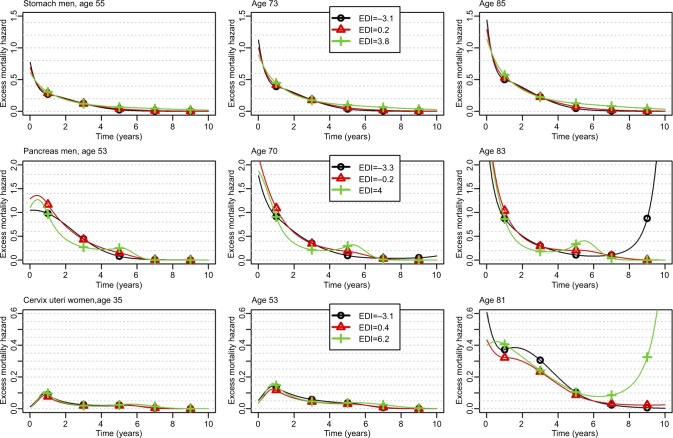
Changes over time since diagnosis of the excess mortality hazard for the 10th, 50th, and 90th percentiles of the age distribution (left, middle, and right column, respectively) and for the 10th, 50th, and 90th percentiles of the EDI distribution (curves with black circles, red triangles, and green crosses, respectively) regarding stomach and pancreas cancers in men, and cervix uteri; patients diagnosed in 2010. **Abbreviation:** EDI, European Deprivation Index.

The changes of the EMH over the time elapsed since diagnosis are given for three values of age and three values of EDI (the 10th, 50th, and 90th percentiles) for 1) LOCP in men, LOCP in women, and melanoma in men (Figure 1); 2) pancreas and stomach in men and cervix uteri (Figure 2); and 3) all other cancer sites (Figures S1–S4). Marked differences were seen by age at diagnosis; the EMHs were higher in old than in young patients, especially during the first year(s) after diagnosis. Changes of the EMH over time since diagnosis illustrate how and when the EDI impact takes place across the follow-up at specific ages at diagnosis and complement the previously given net survival results. For example, the graphs allow illustrating the strong association between the EDI and the EMH for LOCP cancer in both sexes: the curve is always higher in deprived people. A quick look at the graphs might give the false impression that the EHR of the EDI depends on time (see the middle box in Figure 1 where the curves are not parallel for LOCP in women aged 61.5 years). This is because the hazards are proportional on the *log-scale*, whereas the graphs use an *arithmetic scale*. On an arithmetic scale, a value of a time-constant EHR of 2, for example, will display a larger difference between hazards when the baseline hazard is high rather than low.

**Figure 3:**
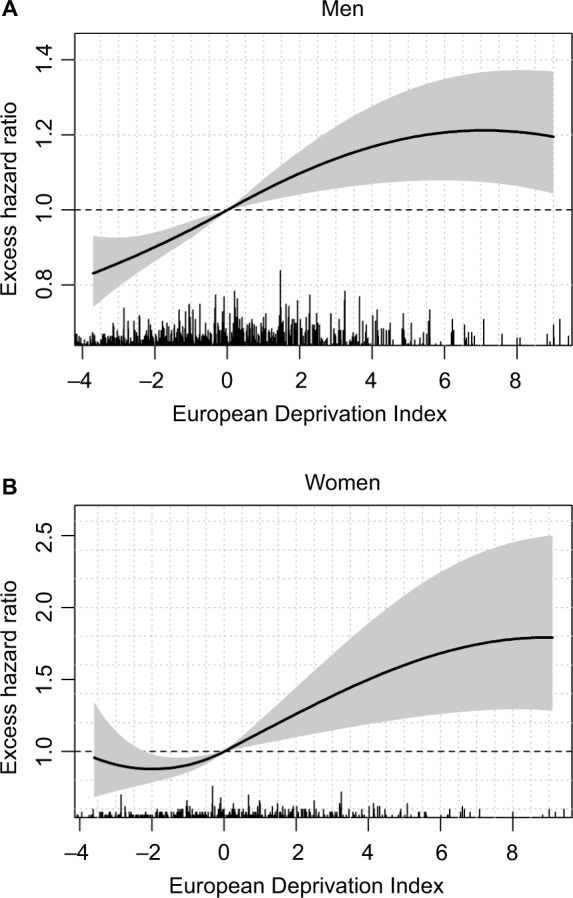
Excess hazard ratio of the EDI on lip–oral cavity–pharynx cancer in men (**A**) and women (**B**) with 95% confidence intervals (shaded area). **Notes:** We limited the EDI values on the x-axis to the 5th and 95th percentiles of the observed EDI distribution in the sex-cancer couple. Rug plots indicate the locations of the observed EDI values. **Abbreviation:** EDI, European Deprivation Index.

For LOCP cancers in both sexes, the model-building strategy retained a nonlinear functional form (though time-constant) for the log-EHR of the EDI (Table S4). In men, the EHR increased according to EDI values but then plateaued in the more deprived people (Figure 3A); however, in women, a plateau is seen in both the least and the most deprived people (Figure 3B).

We also observed a substantial association between the EDI and the EMH in melanoma in men (bottom plots of Figure 1): the retained EHR of EDI was constant in time with a linear functional form (Table S3).

**Figure 4:**
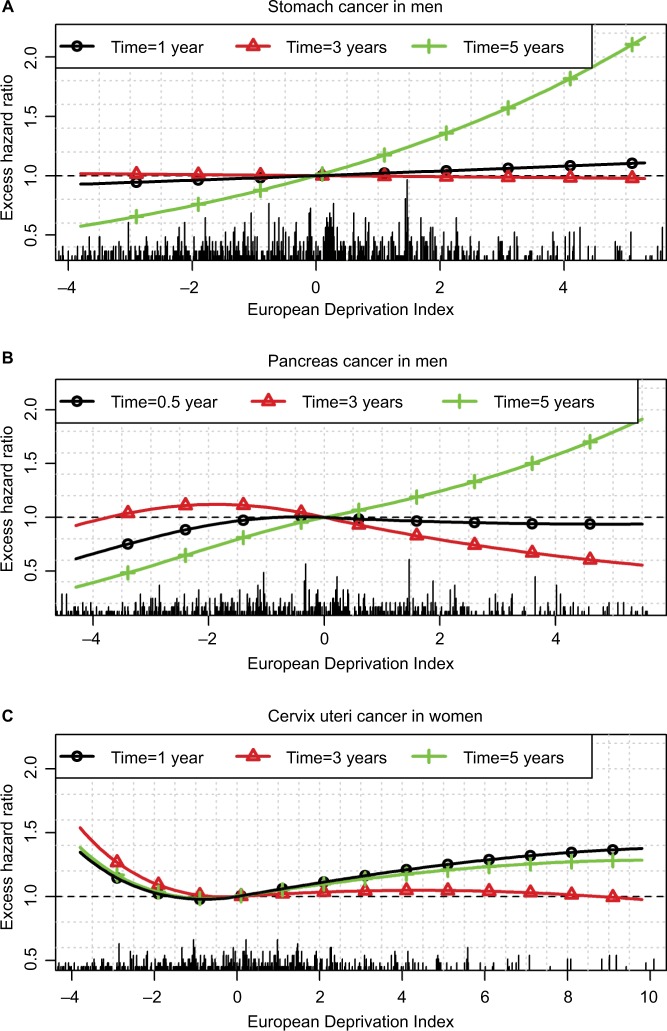
Excess hazard ratio of the EDI at different times after diagnosis for stomach (**A**) and pancreas (**B**) cancers in men and cervix uteri (**C**) cancer in women. **Notes:** We limited the EDI values on the x-axis to the 5th and 95th percentiles of the observed EDI distribution in the sex-cancer couple. Rug plots indicate the locations of the observed EDI values. **Abbreviation:** EDI, European Deprivation Index.

For stomach cancer in men, we observed higher EMHs in deprived patients starting from 5 years after diagnosis (Figure 2, upper plots), and thus, weak differences between Q1 and Q5 regarding 1- and 5-year ASNS (Table 1 and Table S4) but a substantial difference (11%) regarding 10-year ASNS (Table S5). Figure 4A shows the time-dependent EHR of the EDI in stomach cancer, especially a substantial impact in late follow-up (5 years), even if the EMHs are quite low after 5 years (Figure 2).

For pancreas cancer in men, we observed a very complex pattern associated with the EDI, especially a lower EMH within the first year in the less deprived patients vs other patients and, in contrast, a lower EMH between years 1 and 4 in deprived vs less deprived patients. Therefore, the impact of deprivation on net survival was high over the first year after diagnosis and resulted in a substantial difference in 1-year ASNS between the less deprived to the other patients (Table S4). This difference shrunk at 5 years because of the reverse association observed after 1 year (Figure 4B). At 6 months, the EHR is <1 at small EDI values (ie, in the less deprived patients) and ~1 at other values. At 3 years, the EHR is slightly >1 in the less deprived patients and slightly <1 in the more deprived (Figure 4B). At 5 years, the EHR should be interpreted with caution because the prognosis of pancreas cancer at 5 years is rather poor, and thus, the number of patients still at risk is rather low.

Finally, for cervix uteri cancer, the EMH was higher in the less deprived people than in people with a median EDI whatever the time since diagnosis and the age at diagnosis (Figure 2). Therefore, the 1-, 5-, and 10-year ASNS was lower in the less deprived people than in others (Tables 1, S4, and S5). This corresponds to very complex nonlinear and time-dependent EHRs of the EDI (Figure 4C), the main information relying on the U-shape of the curves (ie, EHRs >1 were observed in the least and the most deprived people).

#### General contextual effect

The MEHRs with and without adjustment on the EDI are given in Table S6. For LOCP cancers in men, the median increase in the EMH between similar patients from IRIS with a high vs a low excess mortality was 25.5% before adjustment on the EDI (MEHR=1.255) and 21.4% after adjustment (MEHR=1.214).^35^ The figures in women were also substantial: the median increase in the EMH was 23.8% before adjustment vs 8.1% after adjustment. This reveals an important general contextual effect for LOCP cancers; the EDI seems to explain an important part of EMH variability between IRIS, especially in women. We also observed an important decrease (before vs after adjustment on the EDI) of the MEHR for prostate, melanoma, and pancreas cancers in men (Table S6).

## Discussion

In an international context of increasing socioeconomic inequalities,^38^ describing and quantifying the association between socioeconomic inequalities and the excess cancer-related mortality hazard is important. Here, we used a strategy able to deal with specific methodological requirements: the use of a relevant measure of deprivation and a relevant mortality indicator (the EMH) estimated using a flexible regression model able to deal with nonlinear and time-dependent associations. The approach should account for the fact that individuals within a cluster share similar characteristics and should also allow to summarize the “importance” of the cluster level on the EMH. We applied this approach to 17 solid tumors diagnosed in a specific area of France and followed up over 10 years after diagnosis to investigate the change over time of the excess mortality by age and socioeconomic level. We summarize the recommendations we believe important to describe the association between socioeconomic deprivation and the EMH (Table 2).

Using population-based cancer registry data ensures depicting the full picture of cancer survival inequalities. For decades, the notifications of cancer cases come from many different sources (public and private pathology laboratories and hospital discharge databases as well as databases of the National Health System). Even if the number of data sources has dramatically increased since 1997, it was to collect further information on cancer cases such as treatment, thus not affecting the core of the cancer registry data and their exhaustiveness. For these reasons, we do not suspect any differential ascertainment over the study time period nor between areas of residence or individual and area-level socioeconomic determinants. We used the EDI to quantify the deprivation as this index was built to be reproducible in European countries.^18^ We assumed that 1) the EDI assigned to each IRIS remains constant from 1997 to 2010, and 2) the patient’s deprivation corresponds to the EDI measured at the time of diagnosis (no misclassification). We considered these assumptions reasonable because 1) the crude level of the EDI has little significance per se: it is more the ranking of each IRIS across the overall distribution which is of interest and this ranking is less influenced by time, and 2) the number of patients moving after the diagnosis of cancer, which can be seen as a misclassification problematic, should be low for different reasons (access to cancer treatment, preservation of social network, etc). Bryère et al showed that the bias of such misclassification on the association between deprivation and cancer incidence was minimal in their study context.^39^ However, more research should be conducted in the context of deprivation and cancer survival.

We recommend using flexible parametric regression models and underline the importance of examining the changes of the EMH over time since diagnosis together with the net survival (Figures 1 and 2) and the EHRs (Figures 3 and 4); this ensures relevant and complementary clinical information.^40^–^43^ Indeed, at a given time *t*, the probability of net survival is a cumulative measure up to time *t*, whereas the EMH gives an instantaneous picture of what happens specifically at time *t*. It quantifies the instantaneous rate at which subjects experience an excess death (given they survived up to *t*) and, being a rate, the EMH may be >1. When the EMH is low (say <0.1) and practically constant over the year, its value is very close to the annual probability of death from the disease. With higher values, a back-transformation on the probability scale (using the classical relationship between hazard and survival) may be advantageous for clinical interpretation because it provides a conditional probability.

Caution should be taken when interpreting the changes of the EMH over time because its decrease in a population (“marginal” EMH) could be due either to true decreases of individual EMHs or to a “selection effect” over time.^44^ For example, when a population includes a mix of 1) patients with localized cancer stages and low and constant-in-time EMH and 2) patients with advanced stages and high and constant-in-time EMH, the analysis of this population as a whole (in the absence of information on stage) will estimate a “marginal” EMH that will decrease with time. The more “frail” individuals (with the higher hazards) will die early, whereas the more “robust” individuals (with the lower hazards) will stay at risk (are “not selected to die”): the marginal EMH will then decrease and approach the EMH of the more robust subjects.^44^ Nevertheless, the possibility to estimate and depict those quantities (EMH and EHR) over time using flexible functional forms is an important advantage of our proposed methodology compared to using a simpler model with either shape-restricted baseline hazard (such as monotonic for the Weibull distribution) or assuming proportional hazard ratio. As an illustration, we fitted a simple model without a random effect and assumed a Weibull distribution and linear and proportional hazard ratios for each prognostic factor. We applied this simple model to the LOCP cancer in men and in women, and to pancreas cancer in men. In LOCP cancers, using this simple model would not allow to identify the plateau of the EHR for the most deprived men nor both plateaus for the less deprived and the most deprived women (Figure 3). From this simple model, the estimated EHR comparing women with EDI=4 to women with EDI=0 was 1.21 [1.09;1.34] compared to 1.50 [1.19;1.90] with our approach. Neglecting the time-dependent effect of the EDI for pancreas cancers with the simple model would also lead to a substantial oversimplification, showing no evidence of an association between the EDI and the EMH (EHR for 1-unit increase of the EDI=1.00 [0.98;1.022]), compared to the complex time-varying association found with our approach (Figure 4B).

We advocated the use of a model-building strategy to eliminate spurious time-dependent and nonlinear EHR functions from a flexible regression model. We used the one proposed by Wynant and Abrahamowicz,^36^ but an alternative model-building strategy could be used, such as the one proposed by Royston and Sauerbrei.^45^ However, the development of algorithms for model building is still an active area of statistical research, and studies comparing the ability of model-building strategies to eliminate spurious time-dependent and nonlinear EHR functions would be useful for giving advice to the analyst. Whatever the choice of the model-building strategy, fitting regression models requires observing enough events for providing reliable estimates, and this may be an issue in small sample studies or when studying cancer with a very good prognosis. In our work, we did not analyze some sex-cancer couples because of insufficient observed events for fitting the “full model” based on the “rule” of observing at least 10 events per parameters,^46^ even though this “rule” is still debatable.^47^

We evidenced an association (linear and constant-in-time) between the EDI and the EMH in colon–rectum, lung, melanoma, and prostate cancers in men as in breast cancer in women, with lower survivals in the most deprived. We also found a substantial deprivation gap in LOCP cancers in both sexes with >10% differences in 5-year ASNS between deprivation Q1 and Q5. The main drivers of LOCP cancer are alcohol and tobacco consumptions, and both are associated with other comorbidities; this limits the treatment possibilities and leads to poor prognoses. In France, the prevalence of tobacco smokers in men or women is generally higher in deprived than in most affluent people though women with management responsibilities seem more prone to smoking than others.^48^,^49^ Regarding alcohol consumption, the picture is more complex and differs with sex: excessive alcohol consumption is more frequent among women with management responsibilities vs other women but affects both extremes of the deprivation scale in men.^48^ In addition, the probability of alcohol avoidance is quite high among deprived people.^48^ These observations are in line with the patterns of the EHR of the EDI (Figure 3A and B).

For stomach cancer in men, deprived patients were found exposed to a higher excess mortality at 5 years after diagnosis vs less deprived patients, whereas the EDI plays no role at 1 or 3 years after diagnosis (Figure 4A). This may be due to 1) more comorbidities among deprived patients that may preclude the recourse to the best treatment strategies and lead to higher risks of relapse in the long term and/or 2) lower patient adherence to cancer follow-up among deprived patients. For cervix uteri, we showed a higher excess mortality among the less deprived patients (Figure 4C): it may be linked to a higher participation to cervical screening among the less deprived subjects,^50^ which would eliminate a higher number of curable precancerous lesions in affluent than in deprived people.

The interpretation of such relationships would benefit from additional information on cancer stage at diagnosis and comorbidities. Such data were not available for the present study but French registries have started the systematic collection of stage at diagnosis. Another limitation of the study is the lack of deprivation-specific expected mortality rates in France. Therefore, the use of the general-population mortality as expected mortality rate overestimates the excess hazard in the more deprived people (because their expected mortality is usually higher than the “average” mortality in the general population) and underestimates it in the less deprived ones. This may lead to amplify the impact of the EDI and highlight the urgent need to produce deprivation-specific life tables in France.

In the present article, we predicted the ASNSs from the fitted regression model and obtained the ASNSs even in case of sparse data because model-predicted NSs can be obtained after the date of the last event in a specific stratum (which is another advantage of using our proposed methodology compared to using only nonparametric estimates). However, these predictions rely on the assumption that the regression model is correctly specified. For each sex-cancer couple, we checked the goodness of fit of the model by comparing the model-based ASNS with the nonparametric ASNS as given by the Pohar-Perme estimator^24^ for each deprivation quintile and each period of diagnosis ([1997–2000], [2001–2005], [2006–2010], and all periods combined). Comparing the 5-year ASNSs showed the good accuracy of model-based NS prediction (Figure S5).

Quantities that measure between- and within-cluster variability may help interpreting the results. We extended the median hazard ratio proposed by Austin and Merlo^35^ to our context of EMH to reach a better understanding of the impact of a within-IRIS clustering on the EMH. According to these authors, one would additionally compare the MEHR to the estimated EHR of each prognostic factor. However, in our final explanatory model, we rarely retained a single parameter for each prognostic factor, which makes impossible such a comparison. So, though the MEHR has the merit of simplicity, an interesting perspective would be to extend the approach proposed by Oliveira et al.^51^ These authors derived an intra-class correlation coefficient for time-to-event regression models with a random effect (frailty). As in a linear model, this coefficient is defined as a ratio of variance components, which allows interpreting the coefficient as the proportion of the total variance due to the between-IRIS variability.^52^ However, the approach proposed by Oliveira et al suits their specific models that include closed forms of marginal variance, which leads to closed forms of intraclass correlation.^51^ A future work would check whether their approach may be applied to our model.

Evaluating the interactions between prognostic factors is a further important step when describing the association between deprivation and cancer survival. For example, the interactions allow checking whether the EHR of the EDI is the same whatever the age at diagnosis. In an exploratory analysis that used Royston and Sauerbrei’s methodology^45^ to study interaction, our preliminary results suggested that such interactions do exist for some cancers (results not shown). These results still need a validation of a robust statistical approach to test the interactions. Another important research area would be to extend this analysis by including socioeconomic measures defined at both the individual level and the area level. Indeed, with the EDI being an ecological variable,^53^ the estimated effect of deprivation actually combines individual and contextual effects. Adjusting for both subject- and area-specific measures would allow disentangling individual from contextual effects of deprivation.^16^,^54^–^56^

International comparisons of the association between socioeconomic deprivation and cancer survival are useful to understand differences between health care systems. Several studies have already reported poorer prognoses in deprived vs less deprived cancer patients.^2^,^4^–^6^,^57^ However, comparing the results is difficult because of distinct study designs, statistical analysis methods, and deprivation indexes. We hope the proposed approach will provide a methodological basis for such explorations. The use of the present approach with the EDI in other European countries^20^ will ease comparisons between European health care systems.

## Figures and Tables

**Table 1:** Age-standardized 5-year net survivals by cancer and deprivation quintiles (Q1–Q5, from the less to the more deprived) and EHRs for 1-unit increase of the EDI, in men and women, with their 95% confidence intervals

Cancer	Men						Women					
	Q1	Q2	Q3	Q4	Q5	EHR	Q1	Q2	Q3	Q4	Q5	EHR
LOCP	41 [38;43]	38 [36;40]	35 [33;37]	32 [30;33]	29 [27;31]	NL	55 [49;62]	60 [55;64]	58 [54;62]	52 [48;56]	43 [38;47]	NL
Esophagus	13 [11;15]	12 [11;14]	12 [10;14]	12 [11;14]	11 [10;13]	1 [0.98;1.02]	NA	NA	NA	NA	NA	NA
Stomach	27 [24;29]	26 [24;28]	25 [24;27]	25 [23;27]	24 [21;26]	TD	33 [29;37]	32 [29;36]	32 [29;35]	32 [29;35]	32 [28;35]	1 [0.97;1.03]
Colon/rectum	60 [58;62]	58 [57;60]	57 [56;59]	56 [55;57]	53 [51;54]	1.03 [1.01;1.04]	61 [60;63]	60 [59;62]	60 [59;61]	59 [58;61]	58 [56;60]	1.01 [0.99;1.03]
Liver	17 [15;19]	16 [14;17]	16 [15;18]	15 [13;16]	13 [12;15]	1.01 [1;1.03]	NA	NA	NA	NA	NA	NA
Pancreas	8 [6;11]	4 [3;5]	4 [3;5]	5 [4;6]	7 [5;9]	NL and TD	10 [8;13]	9 [7;12]	10 [8;12]	10 [8;11]	9 [7;11]	1.01 [0.99;1.03]
Larynx	53 [48;57]	52 [48;55]	51 [47;54]	50 [47;53]	50 [46;53]	1.01 [0.98;1.04]	NA	NA	NA	NA	NA	NA
Lung	14 [14;15]	13 [13;14]	13 [12;14]	12 [11;13]	10 [10;11]	1.02 [1.01;1.02]	17 [15;19]	17 [15;18]	16 [14;17]	15 [14;17]	14 [12;15]	1.01 [1;1.03]
Melanoma	87 [84;89]	86 [83;89]	83 [81;86]	81 [79;84]	76 [72;80]	1.08 [1.02;1.14]	91 [89;92]	90 [89;92]	90 [88;91]	89 [87;90]	87 [85;89]	1.04 [0.99;1.1]
Breast	NA	NA	NA	NA	NA	NA	87 [86;88]	86 [85;87]	86 [85;87]	85 [84;85]	83 [82;84]	1.03 [1.02;1.05]
Cervix uteri							55 [51;59]	64 [61;66]	66 [63;68]	65 [63;68]	64 [61;67]	NL and TD
Corpus uteri*							73 [70;75]	73 [70;75]	72 [70;74]	73 [71;75]	73 [71;76]	0.99 [0.96;1.02]
Ovary							39 [36;42]	38 [35;40]	37 [35;39]	36 [34;38]	36 [34;39]	1.01 [0.99;1.03]
Prostate	93 [92;93]	92 [91;93]	91 [90;92]	91 [90;91]	88 [87;89]	1.05 [1.02;1.08]						
Kidney	69 [66;71]	68 [65;70]	67 [64;69]	65 [63;67]	63 [60;66]	1.02 [0.99;1.05]	63 [60;66]	64 [62;67]	64 [62;66]	64 [61;66]	65 [62;68]	0.99 [0.96;1.03]
Bladder*	63 [60;65]	62 [60;64]	60 [58;62]	58 [56;60]	56 [53;58]	1.03 [1;1.05]	55 [50;59]	52 [47;56]	53 [49;57]	52 [48;56]	49 [44;54]	1.01 [0.98;1.04]
CNS	19 [16;23]	19 [15;22]	19 [16;22]	19 [17;22]	19 [16;23]	1 [0.97;1.03]	22 [19;26]	23 [19;26]	22 [19;25]	22 [19;25]	20 [17;23]	1.01 [0.99;1.04]

**Notes:** The ICSS weights were used for standardization. The EHR estimates are adjusted on age at diagnosis and year of diagnosis and based on the final model retained (see Materials and methods section) for cancer with a time-constant coefficient and a linear functional form of the EDI. For cancers with an NL functional form and/or a TD coefficient of the EDI, the EHRs are reported in Figures 3 and 4. The bounds of the deprivation quintiles here correspond to those of the deprivation quintiles observed in the general French population.

* Due to sparse data, we merged age groups ([15–45] and [45–55] into a single group [15–55] [adding up the corresponding ICSS weights]) to calculate the age-standardized net survival.

**Abbreviations:** CNS, central nervous system; EDI, European Deprivation Index; EHR, excess hazard ratio; ICSS, International Cancer Survival Standard; LOCP, lip–oral cavity–pharynx; NA, not available; NL, nonlinear; TD, time-dependent.

**Table 2:** Summary of the guidelines for describing the association between socioeconomic inequalities and cancer survival

Step	Guidelines
Data	
	• Use data from a source that provides an unbiased picture of the whole population, such as population-based registries data
	• Use an appropriate ecological deprivation measure, which can be 1) replicated in other countries (for comparison purposes); and 2) based on as small geographical unit as possible
Method	
	• Define the excess mortality hazard as your main quantity of interest
	• Use general-population lifetables for the expected mortality hazard and the deprivation-specific ones whenever possible
	• Use flexible parametric multivariable regression models, which enable modeling nonlinear as well as time-dependent log excess hazard ratios for prognostic factors (such as the deprivation index)
	• Take account of the multilevel/hierarchical structure of the data to derive correct statistical inference
	• Use a model-building strategy or an information criterion to eliminate spurious nonlinear and time-dependent log excess hazard ratios
Results	
	• Provide model-predicted age-standardized net survivals by deprivation quintile and compare them to the nonparametric estimates (to check the goodness of fit of the model)
	• Give additional and clinically relevant information from the modeling approach: 1) the change with time since diagnosis of the excess mortality hazard for different values of the deprivation index and 2) the excess hazard ratios for the association (eventually nonlinear and/or time-dependent) between the EDI and the excess mortality hazard
	• Quantify the impact of clustering on the excess mortality hazard using the general contextual effect and (whenever possible) an intraclass correlation coefficient

**Abbreviation:** EDI, European Deprivation Index.
